# Diagnosis of *Hepatozoon canis *in young dogs by cytology and PCR

**DOI:** 10.1186/1756-3305-4-55

**Published:** 2011-04-13

**Authors:** Domenico Otranto, Filipe Dantas-Torres, Stefania Weigl, Maria Stefania Latrofa, Dorothee Stanneck, Donato Decaprariis, Gioia Capelli, Gad Baneth

**Affiliations:** 1 Dipartimento di Sanità Pubblica e Zootecnia, Università degli Studi di Bari, Valenzano, BA, Italy; 2 Bayer Animal Health GmbH, Leverkusen, Germany; 3 Istituto Zooprofilattico Sperimentale delle Venezie, Legnaro (PD), Italy; 4 School of Veterinary Medicine, Hebrew University, Rehovot, Israel

## Abstract

**Background:**

*Hepatozoon canis *is a widespread tick-borne protozoan affecting dogs. The diagnosis of *H. canis *infection is usually performed by cytology of blood or buffy coat smears, but this method may not be sensitive. Our study aimed to evaluate the best method to achieve a parasitological diagnosis of *H. canis *infection in a population of receptive young dogs, previously negative by cytology and exposed to tick infestation for one summer season.

**Results:**

A total of 73 mongrel dogs and ten beagles younger than 18 months of age, living in an animal shelter in southern Italy where dogs are highly infested by *Rhipicephalus sanguineus*, were included in this study. In March-April 2009 and in October 2009, blood and bone marrow were sampled from each dog. Blood, buffy coat and bone marrow were examined by cytology only (at the first sampling) and also by PCR for *H. canis *(second sampling). In March-April 2009, only one dog was positive for *H. canis *by cytological examination, whereas in October 2009 (after the summer season), the overall incidence of *H. canis *infection by cytological examinations was 43.9%. Molecular tests carried out on samples taken in October 2009 showed a considerably higher number of dogs positive by PCR (from 27.7% up to 51.2% on skin and buffy coat tissues, respectively), with an overall positivity of 57.8%. All animals, but one, which were positive by cytology were also PCR-positive. PCR on blood or buffy coat detected the highest number of *H. canis*-positive dogs displaying a sensitivity of 85.7% for both tissues that increased up to 98% when used in parallel. Twenty-six (74.8%) out of the 28 *H. canis*-positive dogs presented hematological abnormalities, eosinophilia being the commonest alteration observed.

**Conclusions:**

The results suggest that PCR on buffy coat and blood is the best diagnostic assay for detecting *H. canis *infection in dogs, although when PCR is not available, cytology on buffy coat should be preferred to blood smear evaluation. This study has also demonstrated that *H. canis *infection can spread among young dogs infested by *R. sanguineus *and be present in the majority of the exposed population within 6 months.

## Background

Despite its wide geographical distribution and the fact that it was described in the early 20^th ^century [1], there are still knowledge gaps concerning canine hepatozoonosis caused by *Hepatozoon canis *(Adeleorina: Hepatozoidae), including insufficient understanding of its pathogenesis and the best diagnostic methods to employ for diagnosing this infection. The biological life cycle of *H. canis *in the canine host and its tick vector [1,2] has recently been elucidated in detail [3]. In contrast to other tick-borne protozoa, *H. canis *infects leukocytes and parenchymal tissues and is transmitted to dogs by the ingestion of ticks containing mature oocysts [4]. Following ingestion of infected ticks, sporozoites spread via the bloodstream and lymph to several organs including the spleen, bone marrow, lung, liver and kidney. In these organs, meronts are formed and undergo several cycles of merogony, releasing merozoites, which invade white bloods cells (mostly neutrophils and monocytes) where they form gamonts [3]. The brown dog tick, *Rhipicephalus sanguineus *(Ixodida: Ixodidae), is the main vector of *H. canis *[2,4], although oocysts of this protozoan have also been detected in other tick species feeding on dogs, including *Haemaphysalis longicornis *and *Haemaphysalis flava *in Japan [5] and *Amblyomma ovale *in Brazil [6,7]. *H. canis *is probably one of the most widespread canine vector-borne disease (CVBD)-causing pathogens due to its close association with *R. sanguineus *and the cosmopolitan distribution of this tick species [8,9]. Although large surveys on canine hepatozoonosis are scant [10], a number of reports suggest that *H. canis *infects dogs globally and infections have been reported from four continents [7,10-13].

This protozoan usually causes a chronic infection with relatively mild or no clinical alterations to its host [14,15]. Nonetheless, *H. canis *may also induce severe clinical manifestations (e.g., lethargy, fever, anorexia, weight loss, lymphadenomegaly, and anemia) associated with a high parasite load [16,17]. Concurrent infections may lead to more severe clinical manifestations of hepatozoonosis [18], by impairing the host immune responses [4,19]. Indeed, in endemic areas, CVBD-causing pathogens may infect the same dog with two (*H. canis *and *Ehrlichia canis*) [17], three (*H. canis*, *Babesia *spp., *E. canis*) [10,20] or even four agents (*H. canis*, *Babesia *spp., *E. canis*, *Leishmania infantum*) [21].

The diagnosis of hepatozoonosis is frequently based on the detection of intracytoplasmatic ellipsoidal-shaped gamonts in stained blood smears by microscopy and on the histopathological visualization of meronts and/or monozoic cysts in tissues [22,23]. Nonetheless, serological tests, such as the indirect fluorescent antibody test (IFA), have been developed to detect anti-*H. canis *antibodies [24] with a high sensitivity, mainly in dogs with chronic infections [19].

Molecular diagnosis based on both conventional [25] and real time polymerase chain reaction (PCR) [26], developed during the last decade, greatly contributed to understanding the spread of this protozoan in canine populations. From a practical standpoint, these methods applied on blood were shown to be more sensitive and specific for the diagnosis of this pathogen than other methods [10]. In addition, molecular analysis of target sequences also facilitated the separation of *Hepatozoon americanum *from *H. canis *and its designation as the agent of American canine hepatozoonosis [25,27,28].

Although PCR is considered the most sensitive detection method for canine hepatozoonosis, microscopic examination of blood smears is a simple technique frequently used for the diagnosis of this infection. Nonetheless, few studies have compared these methods [10] and a diagnostic gold standard has not been clearly established. Likewise, information is lacking on the reliability of different tissues for the molecular detection. Finally, little information is available on the incidence of hepatozoonosis in young dogs living in areas where this infection is endemic. Our study aimed to evaluate the best method to achieve a parasitological diagnosis of *H. canis *infection in a population of receptive young dogs, previously negative by cytology and exposed to tick infestation for one summer season. Tissue samples from a selected animal population monitored in a previous study [21] were used and the results of cytology (on whole blood, buffy coat and bone marrow) and of molecular detection (on whole blood, buffy coat, bone marrow and skin samples) were compared. The relationships between the presence of *H. canis *and laboratory parameters were also examined.

## Methods

### Animals and sampling procedures

Dogs enrolled in the study included 73 mongrels and ten beagles younger than 18 months of age that had been sequentially monitored during a field trial over a 1-year period [21]. The sampled population lived in a shelter located in southern Italy where ticks and fleas and the presence of sand flies were recorded in previous entomological studies [29,30]. In March-April 2009 (before the summer season started), all animals enrolled but one were negative for *H. canis *by cytology of blood, buffy coat and bone marrow smears whereas some dogs were positive for other CVBD-causing pathogens as reported elsewhere [31]. The dogs were kept under their usual housing conditions and untreated against ectoparasites from the baseline date (March-April 2009) until the second sampling in October 2009 (after the summer season). Between these two sampling dates, a high level of *R. sanguineus *infestation was recorded in the same dog population [29].

On October 2009, blood, skin tissue and bone marrow were sampled from all of the 83 dogs. The study and the diagnostic procedures were conducted in accordance with the principles of animal welfare and experimentation.

### Cytology

Blood, buffy coat (separated by centrifugation), and bone marrow smears were prepared on glass slides and stained with the MGG Quick Stain (Bio Optica Spa, Italy). Stained-smears were examined under light microscopy for the presence of intracellular inclusions of *H. canis*. Each smear was examined for 10 minutes (100 microscopic fields) under a 100 × oil immersion objective.

### Polymerase chain reaction (PCR)

DNA was extracted individually from buffy coat, bone marrow and blood samples using a commercial kit (Qiagen, Milan, Italy) and from skin samples by using a different DNA purification kit (Gentra Systems, Minnesota, USA), following the manufacturers' instructions. A fragment of the 18S rRNA gene (666 bp in size) was amplified by PCR, using the primers HepF (5'-ATACATGAGCAAAATCTCAAC-3') and HepR (5'- CTTATTATTCCATGCTGCAG-3') [32]. PCR amplifications were carried out in a total volume of 50 μl, including ~100 ng of genomic DNA, 10 mM Tris HCl, pH 8.3 and 50 mM KCl, 2.5 mM MgCl2, 250 μM of each dNTP, 50 pmol of each primer and 1.25 U of AmpliTaq Gold (Applied Biosystems, Foster City, CA, USA). The amplification protocol was employed in a thermal cycler (2720, Applied Biosystems, Foster City, CA, USA) as following: 95°C for 12 min (for polymerase activation), followed by 34 cycles of 95°C for 30 sec (denaturation); 57°C for 30 sec (annealing); 72°C for 1 min and 30 sec (extension), followed by 7 min at 72°C (final extension), as previously described [32]. Negative controls (no DNA template, blood, bone marrow and skin negative reference samples) were included in all PCR reactions. Amplicons were resolved in ethidium bromide-stained agarose (Gellyphor, EuroClone, Milan, Italy) gels (1.5%) and sized by comparison with Gene RulerTM 100-bp DNA Ladder (MBI Fermentas, Vilnius, Lithuania) as molecular marker. Gels were photographed using Gel Doc 2000 (Bio-Rad, Hercules, CA, USA). Amplicons were purified using Ultrafree-DA columns (Amicon, Millipore, Milan, Italy) and sequenced directly (Applied Biosystems, Monza, Milan, Italy) using the Taq DyeDeoxyTerminator Cycle Sequencing Kit (Applied Biosystems, Monza, Milan, Italy). Sequences were determined in both directions (using the same primers individually as for the PCR). Sequences were compared with 18S rRNA gene sequences of *H. canis *available in GenBank.

### Clinical and hematochemical evaluation and categorization

Clinical signs suggestive of *H. canis *infection (e.g., weight loss, pale mucous membranes, and lymphadenomegaly) were recorded in each dog's file at the time of the sampling only. In October 2009, hematological and serum biochemistry parameters including serum proteins were recorded only for 35 of the 83 animals enrolled. Serum protein electrophoresis was carried out by agarose gel electrophoresis and complete blood counts (CBC) were obtained using an automated cell counter (Abbott Cell-Dyn 3700), being the following parameters recorded: hemoglobin concentration (Hb), hematocrit (Hct), nucleated red blood cell count (nRBC), white blood cells count (WBC), platelet count (PLT). Total serum protein (TP), albumin and γ-globulin were also recorded. Alterations in these parameters were assessed in relation to infection by *H. canis *and to clinical signs recorded by the attending veterinarian, at the time of the sampling. Standard canine hematological reference ranges were used for comparison [33].

### Statistical analysis

The prevalence recorded by each test was calculated at both follow-ups. A six-month incidence rate was calculated on the basis of cytology as the proportion of new positive cases divided by the initial population of dogs negative by cytology. The sensitivity of each test was calculated as the proportion of true positives divided by the sum of true positive and false negative dogs. The sensitivity of each test was also calculated in parallel (Multiple test evaluation in WIN Epi). The true positive status of a dog was *a priori *defined as a dog positive to one or more cytology or PCR tests, considering each test used as 100% specific (i.e., there was no possibility of misdiagnosis by cytology as the morphology of *Hepatozoon *is characteristic and also not by PCR as the identity of amplified products was confirmed by sequencing). Agreement among tests performed was evaluated by k statistics and kappa values were ranked as low (0.2 < k < 0.4), moderate (0.4 < k < 0.6), good (0.6 < k < 0.8), or excellent (k > 0.8). The software used was SPSS for windows, version 13.0 (SPSS Inc., Chicago, IL) and WinEpiscope 2.0 [34].

## Results

**Figure 1:**
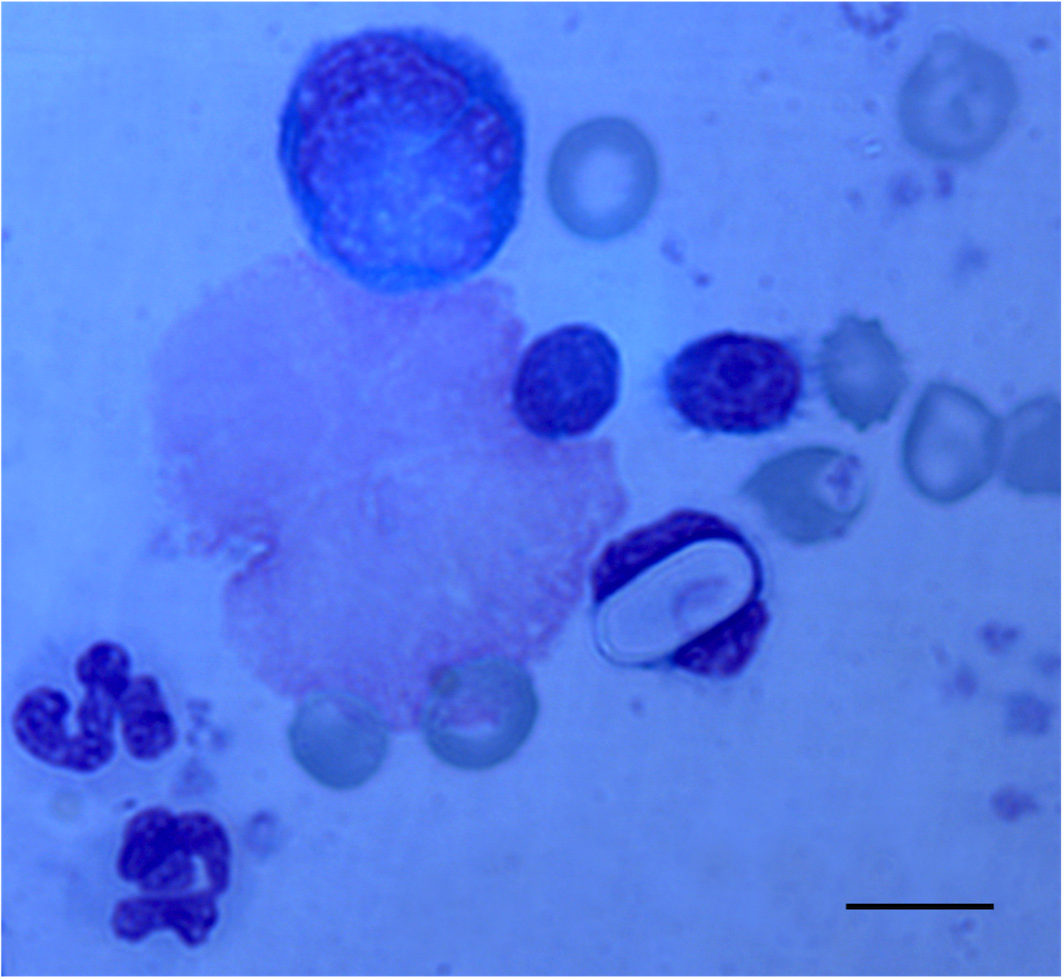
**Cytology of buffy coat-stained smears**. Buffy coat-stained smear showing an ellipsoidal-shaped gamont of *Hepatozoon canis *in the cytoplasm of a leukocyte. Scale bar = 10 μm.

In March-April 2009, out of the 83 animals enrolled and tested for *H. canis *by cytology on whole blood, buffy coat and bone marrow, only one (1.2%) was positive on bone marrow. On October 2009, after the summer season, the cytological examinations (Figure 1) of the same animals showed a positivity rate ranging from 10.8% (blood) up to 41.5% (buffy coat, Table 1) and the total percentage of animals positive by one or more cytological tests reached up to 44.6% (data not shown). This led to an overall incidence of *H. canis *infection inferred exclusively by cytology positivity to one or more tests of 43.9%. The results of the molecular tests carried out on October 2009 showed a higher number of animals positive by PCR (from 27.7% up to 51.2% on skin and buffy coat, respectively), with an overall positivity of 57.8% (Table 1). At the BLAST analysis the sequenced amplicons were identical with those of *H. canis *available in GenBank (AY461378, AF176835). No significant differences in *H. canis *infection rate were recorded between the mongrel dogs and beagles.

**Table 1:** Number and percentage of dogs positive to *Hepatozoon canis *on October 2009 by cytology of blood, buffy coat and bone marrow or by PCR on blood, buffy coat, bone marrow and skin.

Tissues	**Cytology **^**a**^	**PCR **^**a**^
Blood	9/83 (10.8)	42/83 (50.6)
Buffy coat	34/82 (41.5)	42/82 (51.2)
Bone marrow	13/80 (16.3)	39/81 (48.1)
Skin	-	23/80 (27.7)

^a ^Positive/tested (%)

By combining all the cytological and molecular tests, 59% (49/83) of the dogs were found to be infected by *H. canis *after the summer season. All dogs that were positive by cytology were also positive by PCR, except one. The majority of infected animals (n = 33; 67.7%) were positive by 3 (n = 12), 4 (n = 11) or 5 (n = 10) cytological and/or molecular tests simultaneously with a few being positive by one or two tests (n = 6; 7.2%), or by six or seven tests (n = 10; 12%) (data not shown). In addition, 66.6% (n = 10) of the animals positive by two or three cytological tests were also positive by PCR on all the tissues examined.

PCR on blood or buffy coat proved to be the most sensitive assays thus able to detect the highest number of *H. canis *positive individuals (Table 2). In contrast, PCR on skin showed the lowest sensitivity. Interestingly, the likelihood of finding positive results on the skin samples increased with the higher number of other positive tests from the same dog (χ^2 ^= 46.78; p < 0.01). Thus, skin PCR positivity is most likely linked to a disseminated state of the infection in the dog's body.

**Table 2:** Sensitivity of cytology and PCR on blood, buffy coat and on bone marrow and PCR on skin for *Hepatozoon canis*.

Test	Sample	Sensitivity (95% CI)
Cytology	Blood	18.4% (7.52-29.2)^a,c,d,g,l^
	Bone marrow	28.3% (15.2-41.3)^b,e,h,m^
	Buffy coat	69.4% (56.5-82.3)^a,b^
PCR	Skin	50% (35.5-64.5)^c,f,h,I,n^
	Bone marrow	83% (72.2-93.7)^d,e,f^
	Blood	85.7% (75.9-95.5)^g,h,i^
	Buffy coat	85.7% (75.9-95.5)^l,m,n^

* Significant differences are marked by equal letters.

Overall, molecular detection on all tissues but skin, had a higher sensitivity than cytology (Table 2). Indeed, PCR on both blood and buffy coat showed the highest sensitivity (85.7%) whereas the cytology on blood had the lowest (18.4%). In particular, when comparing the sensitivity of PCR with the different tissues, PCR on buffy coat, blood and bone marrow was more sensitive (p < 0.05) than on skin. The agreement of the tests was never excellent, but was good between cytology and PCR on buffy coat (0.7) and among all the PCR tests (ranging from 0.7 up to 0.8), except on skin (data not shown). Again, when PCR on buffy coat and blood were used in parallel, the sensitivity increased up to 98%. On the molecular examination of cytology-negative dogs, bone marrow PCR detected the highest number of positive samples (23.9%) followed by buffy coat (22.2%), blood (21.7%) and skin (8.6%). Out of 49 dogs positive for *H. canis*, 19 were co-infected with one (11 dogs) or more pathogens (8 dogs) (see Table 3).

**Table 3:** Number and percentage (in brackets) of animals infected by *Hepatozoon canis *and coinfected with one or more pathogens (data from ref. [21]).

Pathogen	Number of animals (%)
*Anaplasma platys*	8 (16)
*Ehrlichia canis*	2 (4)
*Bartonella *sp.	1 (2)
*A. platys *and *Leishmana infantum*	3 (6)
*A. platys *and *Babesia vogeli*	1 (2)
*A. platys *and *E. canis*	1 (2)
*B. vogeli *and *E. canis*	1 (2)
*A. platys*, *B. vogeli *and *L. infantum*	1 (2)
*A. platys*, *B. vogeli*, *L. infantum *and *Bartonella *sp.	1 (2)

Out of 49 dogs positive for *H. canis*, 19 were co-infected with one (11 dogs) or more pathogens (8 dogs).

None of the dogs showed apparent clinical signs at the sampling time, irrespective of their positivity for *H. canis *by one or more cytological and molecular tests. However, 26 (74.8%) out of the 28 animals positive for *H. canis *showed hematological abnormalities with absolute eosinophilia being the commonest alteration recorded (20/26), followed by leukocytosis (12/26), lymphocytosis (8/26), neutrophilia (6/26), monocytosis, thrombocytopenia (5/26) and anemia (1/26). Hematological alterations occurred both in dogs infected exclusively with *H. canis *(n = 13) and those co-infected with other CVBD-causing pathogens (n = 13).

## Discussion

By the comparison of cytological examination on different tissues before and after the summer season, a high incidence of *H. canis *infection (43.9%) was recorded in the population of young dogs examined in this study. Indeed, the cytology of buffy coat and blood smears is routinely used for the diagnosis of canine hepatozoonosis. If it were possible to calculate an incidence rate based on PCR with comparison between March-April 2009 vs. October 2009, it would be expected that the incidence rate would have been even higher than the rate based on cytology, as the sensitivity of PCR proved to be considerably higher than that of cytology. Little information is available in the literature on the incidence of *H. canis *infection in pups and young dogs and thus data presented here are of interest in indicating that this infection could spread quickly among young dogs and be present in the majority of the exposed population. The high prevalence of infection detected in October 2009, soon after the summer season, indicated that the infection was transmitted to a large proportion of the dog population studied, which fits with data showing that the highest *R. sanguineus *population density occurred during the summer months in the same dog population [29]. In previous studies, the prevalence of infection inferred by blood smear cytology varied from 1% [35] up to 39.2% [36], being much higher in some studies using molecular tests (up to 63.8%) [37]. Accordingly, the molecular tests employed in the current study detected a higher proportion of positive animals (57.8%) than that diagnosed by combined cytology of several sample types (44.6%). Overall the results of the cytological and molecular tests in diagnosing of *H. canis *infection overlapped due to the fact that animals most likely had a recent infection, as also inferred from both the time of sampling collection (soon after the period of the highest tick population density) and the young age of animals. It is likely that a long time gap between the initial infection and the date of testing for it will increase the probability that cytological examination might fail in detecting low or intermittent parasitemia, thus resulting in false negative results. This suggests that when no information is available on the date of potential infective tick exposure, PCR on either blood or buffy coat should be preferred to cytology for the diagnosis of *H. canis *infection.

The combination of PCR on all four samples (blood, buffy coat, bone marrow and skin) was able to detect 13% more of positive dogs when compared to PCR on buffy coat alone. This increased sensitivity justifies PCR on multiple tissues and not only a single one when searching for *H. canis *infection in a suspected dog.

Cytological detection of *H. canis *in buffy coat smears is certainly recommended over examination of a blood smear, as it is 3.8 times more sensitive, in agreement with a previous study [38] and also 2.5 times more sensitive than bone marrow cytology. A combination of cytological examination of blood, buffy coat and bone marrow smears allowed the detection of only 7.5% more samples than buffy coat alone, and therefore it might not be justified to sample the bone marrow of suspected dogs, if a buffy coat smear can be examined.

Although no apparent clinical signs were directly related to *H. canis *infection at the time of sampling, 26 of the *H. canis *infected animals showed hematological abnormalities eosinophilia being the most common alteration observed, followed by leukocytosis, lymphocytosis, neutrophilia, monocytosis and thrombocytopenia. These alterations, in particular eosinophilia, occurred either in animals with single *H. canis *infection or co-infected with other CVBD-causing pathogens. In the latter case, *H. canis *may complicate the panel of clinical alternations related to other pathogens [39]. This is of relevance in geographic areas were CVBD-causing pathogens occur simultaneously in the same individual dog, since it might result in complex disease manifestations in sick dogs, impairing the achievement of a definitive diagnosis and selection of proper therapeutic agents.

## Conclusions

The results presented here suggest that the PCR on buffy coat and blood is the most sensitive assay for the detection of *H. canis *infection in dogs. This technique may be used also as an epidemiological tool for studies in areas were canine hepatozoonosis is endemic or where it is suspected. However, when PCR is not available for immediate testing (e.g., in most of the routine veterinary practices), cytology on buffy coat should be preferred to blood smear evaluation as indicated. This study has also demonstrated that *H. canis *infection can spread rapidly among young dogs infested by *R. sanguineus *ticks and be present in the majority of the exposed population within 6 months. Finally, the achievement of a prompt diagnosis of hepatozoonosis is pivotal in geographic areas were other CVBD-causing agents occur in order to reduce the clinical effects of simultaneous pathogen infections and to select the best therapeutic drug.

## Competing interests

The authors declare that they have no competing interests.

## Authors' contributions

DO conceived the research, collected samples, contributed with data analysis and interpretation and wrote the first draft of the manuscript. FDT and DD contributed with data analysis, cytological exams and interpretation and revision of the manuscript. MSL and SW run the molecular assays and contributed with data analysis and interpretation. GC, GB and DS contributed with data analysis and interpretation and revision of the manuscript. All authors read and approved the final version of the manuscript.
